# Hemorrhage from Umbilical Cord Ulceration Identified on Real-Time Ultrasound in a Fetus with Duodenal Atresia

**DOI:** 10.1155/2019/2680170

**Published:** 2019-02-13

**Authors:** William M. Curtin, Jaimie L. Maines, Christina T. DeAngelis, Niamh A. Condon, Serdar H. Ural, Karmaine A. Millington

**Affiliations:** ^1^Division of Maternal-Fetal Medicine, Penn State University Milton S. Hershey Medical Center, Penn State College of Medicine, Hershey, Pennsylvania, USA; ^2^Department of Pathology & Laboratory Medicine, Penn State University Milton S. Hershey Medical Center, Penn State College of Medicine, Hershey, Pennsylvania, USA; ^3^Department of Obstetrics & Gynecology, Penn State University Milton S. Hershey Medical Center, Penn State College of Medicine, Hershey, Pennsylvania, USA; ^4^Department of Radiology, Penn State University Milton S. Hershey Medical Center, Penn State College of Medicine, Hershey, Pennsylvania, USA; ^5^Department of Pathology, Perinatal Pathology, North well Health/Cohen Children's Medical Center/Long Island Jewish Medical Center, New Hyde Park, New York, USA

## Abstract

Umbilical cord ulceration has been associated with congenital upper intestinal (duodenal or jejunal) atresia and can lead to fatal fetal intrauterine hemorrhage. We report a case of spontaneous hemorrhage from the umbilical cord, incidentally noted at the time of ultrasound in a 33-week fetus with suspected duodenal atresia, in which immediate delivery resulted in a good outcome. Despite many reports in the literature of congenital upper intestinal atresia and its association with umbilical cord ulceration, the propensity for this lesion for fetal hemorrhage, and the resulting perinatal morbidity and mortality, there appears to be a gap in the dissemination of this knowledge. In fetuses with suspected congenital upper intestinal atresia, recognition of the entity of umbilical cord ulceration may be improved by ultrasound with special attention to the amount of Wharton's jelly within the cord. Routine antepartum fetal surveillance may reduce perinatal morbidity and mortality from this condition. A high index of suspicion is needed to make the diagnosis of umbilical cord ulceration in association with congenital upper intestinal atresia. The role of amniotic fluid bile acids in the genesis of this disorder needs further study.

## 1. Introduction

Umbilical cord ulceration is an unusual lesion characterized by degenerative changes in Wharton's jelly and overlying amnion, thinning of vessel walls, and superficially located, friable vessels prone to hemorrhage. This lesion, its association with congenital upper intestinal (duodenal or jejunal) atresia, and its association with increased perinatal morbidity and mortality have been well documented in the literature; despite this, awareness of this condition and the potential associated lethality remains limited [1]. Because of spontaneous and catastrophic intramniotic bleeding, fetal exsanguination and stillbirth can occur in a high frequency of cases. We describe a case of umbilical cord ulceration in a 33-week fetus with suspected duodenal atresia where active intramniotic hemorrhage from the umbilical cord was identified during real-time ultrasound imaging that led to a prompt delivery and a good outcome.

## 2. Case

A 27-year-old nullipara presented to her local hospital at 33 weeks' gestation with decreased fetal movement, uterine contractions, and possible leakage of fluid from the vagina. Testing confirmed rupture of membranes; therefore, the patient was started on antibiotics to increase latency and was given betamethasone to hasten fetal lung maturity. An ultrasound at the community hospital showed polyhydramnios and a fetal double bubble sign consistent with duodenal atresia. The mother was transferred to Penn State Milton S Hershey Medical Center for anticipation of preterm delivery in a fetus that would require postnatal surgery.

**Figure 1:**
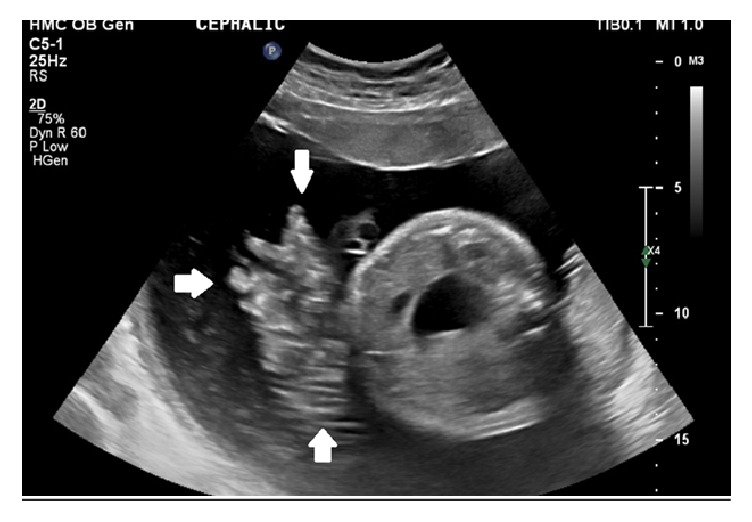
Ultrasound image of fetal blood swirling (arrows) into the amniotic fluid from the umbilical cord adjacent to a transverse view of the fetal abdomen/pelvis.

After transfer, fetal monitoring showed normal fetal heart rate variability with accelerations and occasional decelerations related to contractions. An ultrasound confirmed the double bubble sign and polyhydramnios. Blood was observed in real-time swirling into the amniotic fluid from the umbilical cord (Figure 1). The patient was taken to the operating room and an emergent cesarean section was performed. The amniotic fluid was grossly bloody. A viable male infant weighing 2295 grams was delivered with Apgar scores of 7 and 8 at one and five minutes, respectively.

**Figure 2:**
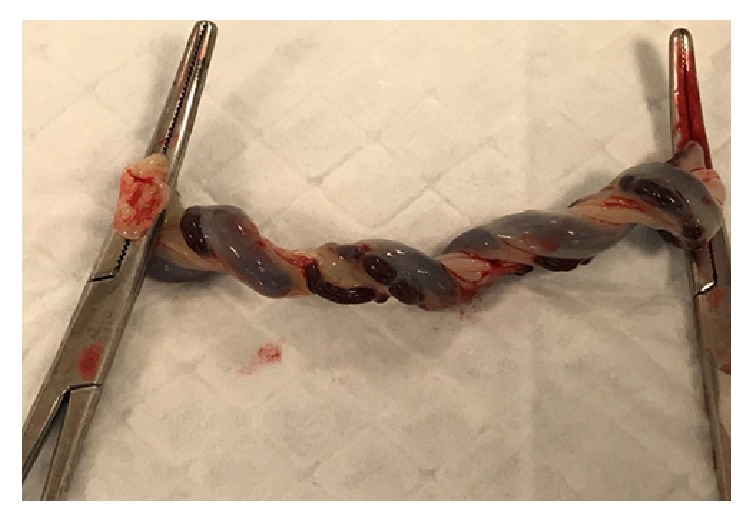
Gross photograph of clamped segment of umbilical cord demonstrating segments of exposed spiraling umbilical arteries.

**Figure 3:**
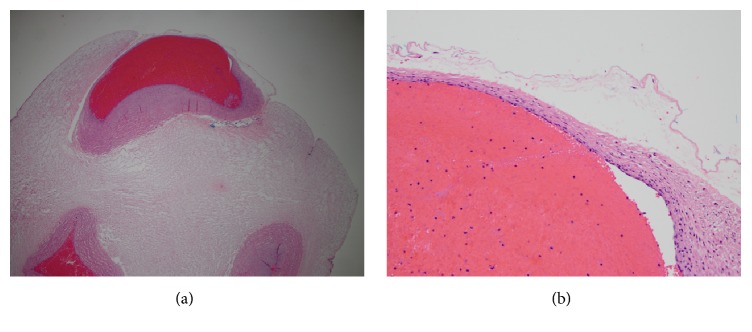
(a). Low power photomicrograph of section of umbilical cord showing superficial location of the umbilical artery (top) with absence of overlying Wharton's jelly, attenuated vascular media, and degeneration of the amnion. This is in contrast to the umbilical vein (lower left) and other umbilical artery (lower right) both of which were completely surrounded by Wharton's jelly and amnion (complete views not in the field). H &E, X 20. (b). High power photomicrograph of ulcerated umbilical artery demonstrating markedly thinned media, absent Wharton's jelly, and degeneration of the overlying amnion. H &E, X 200.

The umbilical cord overall length was 29.5 cm and there were 13, 0.5- 1 cm exposed segments of the umbilical arteries spiraling along the length of the cord (Figure 2). A discrete area from which the hemorrhage emanated was not identified. The placental disk was of normal weight and appeared grossly normal. Histopathologic examination of the cord showed absence of Wharton's jelly covering the umbilical artery, extreme attenuation of the media in the portion of the vessel exposed to the amniotic fluid, and degeneration of the overlying amnion (Figures 3(a) and 3(b)).

The infant had no stigmata of Down syndrome. He appeared pale at birth and an initial hematocrit was 29.4% that was treated by a blood transfusion upon admission to the neonatal intensive care unit. Postnatally the abdominal X-ray revealed air in the stomach and first portion of the duodenum with the remainder of the abdomen appearing gasless, consistent with duodenal atresia. A nasogastric tube was placed and 37 ml of bloody secretions were suctioned. The infant underwent laparotomy on the second day of life where an atresia was noted in the third portion of the duodenum and duodenojejunostomy was performed. He did well postoperatively and was discharged on day 30 of life.

## 3. Comment

The association between fetal hemorrhage from umbilical cord ulceration and congenital intestinal atresia was first noted in 1991 by Bendon et al. [2] when they reported three cases of intestinal atresia associated with linear ulcerations of the umbilical cord. Two of the cases occurred in fetuses that were delivered by cesarean section for fetal distress and the infants were found to be anemic; the third was a stillbirth with hydrops, in the latter the authors presumed secondary to fetal anemia. Since that time, numerous cases of umbilical cord ulceration have been reported in association with upper intestinal atresia. Globally, a large number of cases have been documented in Japan [3]. Only one prior case reported capturing the hemorrhage as it occurred during real-time ultrasound [4]. As in our case, nonreassuring fetal heart rate patterns were noted prior to the ultrasound and prompt cesarean delivery of an anemic infant resulted in a good outcome.

The frequency of umbilical cord ulceration and fetal hemorrhage in fetuses with congenital upper intestinal atresia is estimated to be 15-20% [1, 5, 6]. The typical clinical presentation is preterm labor, nonreassuring fetal heart rate monitoring, and delivery of an anemic infant. In one literature review of 38 cases there was a 32% incidence of fetal demise, 32% incidence of postnatal death, and an overall survival rate of 37% [1]. Macroscopic features of cord lesions in umbilical cord ulceration are variable and may include ulcers that are single or multiple, linear, helical, or punctate. As in our case, other features include visibly exposed umbilical arteries that upon histologic examination show absent Wharton's jelly, thinned or necrotic vessel walls, and overlying necrotic amniotic epithelium. The area of rupture and hemorrhage from the vessels may not be apparent.

The etiology of umbilical cord ulceration in conjunction with congenital upper intestinal atresia is not precisely known. Bendon et al. [2] addressed this association with three hypotheses: (1) vascular reactivity and ischemia leading to the intestinal atresia and the cord ulcer, (2) a primary epithelial cell abnormality in both cord and intestine, and (3) regurgitation of gastric or duodenal contents into amniotic fluid that cause the cord ulceration. Ichinose et al. [6] performed histological examination of the umbilical cord in 20 cases of duodenal or jejunal atresia, only 3 of which were clinically diagnosed, i.e., presented with fetal hemorrhage secondary to umbilical cord ulceration, and compared them to 28 controls. They devised a grading scheme to characterize the degree of ulceration: grade 0, no ulceration; grade 1, loss of amniotic epithelium; grade 2, detachment of basal lamina of amniotic epithelium; grade 3, loss of Wharton's jelly with widespread grade 2 change; and grade 4, exposed umbilical artery or vein. They found that 28/28 controls had no or grade 1 ulceration as compared to 16/20 duodenal or jejunal atresia cases that had grade 2 or greater ulceration. They concluded that these findings suggested gradual pathologic changes in the umbilical cords of fetuses affected by upper intestinal atresia that precede clinical presentation.

Substances that could cause the umbilical cord ulceration in upper intestinal atresia might include gastric acid, digestive enzymes and bile. It is estimated that 85% of duodenal atresia cases are located distal to the ampulla of Vater and the other 15% proximal [7]. In the atresia noted distal to the ampulla of Vater, gastric acid, digestive enzymes, and bile would be anticipated to reflux into the amniotic fluid, while in the atresia located proximally only gastric acid would reflux. Ichinose et al. [6] reported eight cases of duodenal atresia with obstruction proximal to the ampulla of Vater; four of the eight had grade 3 ulcerations of the umbilical cord, suggesting that gastric contents alone may be sufficient to cause ulceration. Their study also reported three fetuses with upper intestinal atresia that presented with nonreassuring fetal status during routine fetal heart rate monitoring and were delivered by emergency cesarean section; however, all three died in the neonatal period and all had intestinal obstruction distal to the ampulla of Vater. These findings suggest that clinically apparent ulceration of the umbilical cord may be more likely in the fetus with duodenal atresia distal to the ampulla of Vater and provides further support that substances from the hepatobiliary tree may have the corrosive effect upon the umbilical cord. Trypsin and bile acids, assayed from amniotic fluid from a 33-week fetus with duodenal atresia, were found to be elevated when compared to fetuses without duodenal atresia, and could be useful markers for diagnosis of the condition [8]. Ohyama et al. [5] suggested that measurement of total bile acids in amniotic fluid may identify fetuses at high risk for umbilical cord ulceration.

The potential role of bile acids in the causation of umbilical cord ulceration is further supported by the potentially corrosive effect that meconium can have on umbilical cord vessels. Meconium contains bile acids similar in composition to those from the fetal gallbladder [9]. Meconium has been associated with vascular myonecrosis of the umbilical cord and umbilical cord ulceration [10]. Segmental hemorrhage from the umbilical cord has been documented in a term pregnancy with suspected prolonged meconium stained amniotic fluid [11]. Complete dissolution of Wharton's jelly associated with exposure of coiled vessels from a 34-week fetus with meconium staining has been documented [12]. Cases such as these lend support to the theory that bile acids may have a toxic effect upon the structural integrity of the umbilical cord.

The lesson learned from our case report was that, despite previous reporting of the association between fetuses with suspected upper intestinal atresia and umbilical cord ulceration, we were unaware of this association and its attendant fetal morbidity and mortality. It is likely that others are also unaware of this association and there is a need to disseminate this information. Pregnancies complicated by suspected congenital upper intestinal atresia may need to have management altered to address the increased risk of fetal hemorrhage. Ultrasound assessment of cross-sectional areas of the umbilical cord with calculation of the area occupied by the Wharton's jelly [13] could be useful to detect asymptomatic umbilical cord ulceration in fetuses with congenital upper intestinal atresia. Routine antepartum surveillance with fetal nonstress testing may be important for all fetuses with suspected upper intestinal atresia. Many of the cases of hemorrhage from umbilical cord ulceration have come to clinical attention when the mother presents with preterm labor and is found to have nonreassuring fetal testing. The clinician will need to have a high index of suspicion for the diagnosis of umbilical cord ulceration and fetal hemorrhage when a patient presents with this clinical scenario. Further studies on whether measurement of total bile acids in amniotic fluid obtained via amniocentesis are needed to determine if there is a discriminatory level for umbilical ulceration risk. For now, efforts are needed to raise awareness of the potential for fatal fetal hemorrhage from umbilical cord ulceration in fetuses with congenital upper intestinal atresia.
